# Sodium Trimetaphosphate Crosslinked Starch Films Reinforced with Montmorillonite

**DOI:** 10.3390/polym15173540

**Published:** 2023-08-25

**Authors:** Konstantinos Noulis, Theofilos Frangopoulos, Athanasia Arampatzidou, Lazaros Tsekmes, Anna Marinopoulou, Athanasios Goulas, Vassilis Karageorgiou

**Affiliations:** Food Process Engineering Laboratory, Department of Food Science and Technology, International Hellenic Univeristy, P.O. Box 141, 57400 Thessaloniki, Greece

**Keywords:** starch films, sodium trimetaphosphate, montmorillonite

## Abstract

Synthetic polymers are the main food packaging material, although they are nonbiodegradable and their recycling process is expensive. A biodegradable, eco-friendly material, with high availability and low cost, such as starch, is a promising solution for the production of films for food packaging. To enhance starch film mechanical and barrier properties, nanoclays have been incorporated within the film matrix. Crosslinking is a well-established method to modify starch properties, but it has not been investigated in combination with nanoclay addition. In the present study, films were developed with starch that was crosslinked through the addition of 5, 15, and 40% wt. sodium trimetaphosphate (STMP) based on dry starch weight. To investigate the interaction between crosslinking and nanoclay addition, montmorillonite (MMT) was added at a 10.5% wt. concentration based on dry starch weight. Experimental data revealed a synergistic effect between STMP crosslinking and MMT addition regarding film thickness, elongation at break, color properties, and opacity. Regarding barrier properties, MMT addition negated the effect of STMP crosslinking, while, in the case of moisture content, it did not alter the effect of STMP crosslinking. Finally, in the case of tensile strength, a synergistic effect followed by a negative interaction was observed. In conclusion, the addition of MMT can potentially enhance, alongside crosslinking, some properties of the films, while other properties are not affected any more than just by crosslinking.

## 1. Introduction

The use of petrochemical plastics is a continuously growing concern, both from an economical and an ecological point of view [1]. Τhe global production of plastics reached 360 million tons in 2019, where half of them were products that would eventually be disposed of, and generally, various industries prefer synthetic plastics due to their physicochemical properties as well as their economic viability [2].

It is, therefore, deemed necessary for environmental reasons to search for an alternative solution for the total or partial replacement of synthetic polymers. Thus, the development of edible and/or biodegradable films, mainly based on polysaccharides and proteins, is promoted [3]. Starch is the predominant choice between polysaccharides to create such a membrane, due to its high availability, high extraction efficiency, and nutritional value, low cost, biodegradability, biocompatibility, and sustainability. Starch-based films exhibit excellent optical (transparent, colorless), organoleptic (odorless, tasteless) and barrier properties (O_2_ and CO_2_ permeability) [2].

On the other hand, there are some important limitations that do not allow the extended use of starch-based films. These films present disadvantages in terms of water vapor permeability and mechanical properties, mainly due to their hydrophilic behavior [4]. Another drawback concerns the mechanical properties, which are considered poor [5] and often easily affected by changes in the environment such as humidity, temperature, and pH [6].

Several attempts have been made over the years, in order to improve the existing ones and even offer some new properties to the starch-based films by adding compounds, such as natural plasticizers, nanofillers, crosslinkers, etc. Even though amylose is mainly responsible for the film-forming capacity of starch-based films, in the absence of a plasticizer the films tend to be brittle, hence a plasticizing agent is usually added, since it helps to extend, dilute and soften the structure, and therefore increase the chains mobility [7]. An often-used plasticizer for starch films is glycerol, which is compatible with amylose and enhances mechanical properties since it affects amylose packing and decreases the forces between molecules [7].

A wide range of nano-sized fillers (at least one dimension in the nanometer range of 1–100 nm) have been tested in the past as additives in plasticized starch, such as phyllosilicates, polysaccharide nanofillers, carbonaceous nanofillers, and many more, all of which differ in geometry and surface chemistry [8]. According to Chung et al. (2010), montmorillonite (MMT) is the most commonly used natural clay that has been applied with success in numerous nanocomposite systems [8]. The structure of montmorillonite consists of an octahedral sheet of alumina or magnesia, surrounded by two tetrahedral sheets of silica [9]. This aluminosilicate clay has a high specific surface area and aspect ratio, it exhibits good adsorption, cation exchange, and drug-carrying capabilities, followed as well by its low cost [9,10]. The addition of nanofillers such as MMT, offers the ability to improve the mechanical, thermal, and barrier properties of films, as well as acquire other useful functions, such as the antimicrobial effect [11].

Crosslinking is a commonly used method of chemical modification, in order to improve starch properties by adding intra- and inter-molecular bonds at random locations [12]. This chemical crosslinking of starch provides several advantages, such as improved stability to water and heat, resistance at low pH, shear and stirring, and formation of a strong and rigid film matrix [8,13]. Sodium trimetaphosphate (STMP) is a safe and non-toxic crosslinking agent, suitable for physical polymers such as polysaccharides, yet crosslinking of a composite biopolymer film has seldom been investigated [14]. STMP is a cyclic triphosphate, obtained by the condensation of phosphoric acid and pyrophosphate at high temperatures [15]. As referred to in Sharma et al. (2020), STMP crosslinking of biodegradable starch films resulted in an increase in the tensile strength, opacity, and solubility and a decrease in water vapor permeability [5].

It is a well-known fact that either STMP crosslinking or MMT addition, according to previously shared papers, could potentially enhance the properties of starch-based films. However, to the best of our knowledge, no information is available on the combined effect of STMP crosslinking and MMT addition on the properties of starch-based films. Furthermore, the available work in the literature, on the crosslinking of lentil starch films is scarce, especially using STMP and not a more common crosslinking agent such as citric acid. The use of lentils as a starch source is an example of how a common legume can be used as a food packaging material, suggesting a potential application for legumes that are unfit for human consumption and are rejected by the food industry. Therefore, the objective and the novelty of the present study was to investigate the effect of different levels of STMP crosslinking of lentil starch, particularly at greater levels than the ones mentioned in the literature, and the effect on the production of films on the absence and presence of MMT, searching for any synergistic interaction between STMP and MMT.

## 2. Materials and Methods

### 2.1. Materials

Glycerol was purchased from Carlo Erba Reagents (Cornaredo, Italy), sodium trimetaphosphate (STMP) from Sigma-Aldrich (St. Louis, MI, USA), NaOH from Lach-ner (Neratovice, Czech Republic) and montmorillonite (MMT) from Nanocel LG (Punjab, India).

### 2.2. Extraction of Starch from Lentils

Starch was isolated from lentils at the pilot plant scale according to the following procedure. Dry lentil seeds were soaked in excess of a sodium metabisulfite 1.5% wt. solution in soft water (≈10 μS/cm) for 48 h at ambient temperature (18–22 °C). Subsequently, the impregnated seeds were separated from the metabisulfite solution and wet ground to a pulp using a hammer mill (APEX 314S-SS, APEX Construction LTD, UK) fitted with a 2 mm screen. During grinding, soft water on a 4:1 weight ratio to the soaked lentils was added. Lentil peals were concomitantly separated from the legume puree through a refining 0.5 mm screen skin and seeds separator/finisher (Gallia, Henri Biaugeaud, France). Congo red dye was used to identify the damaged starch granules and the results showed that the damage was insignificant. Following skin removal, the lentil pulp was wet-sieved through a custom-made vibrating sieve-separator where smaller fiber, flesh, and skin pieces were removed through subsequent 0.5 mm and 53 μm sieves. Finally, starch was isolated and purified through 4 successive sedimentation steps in water, where the impurities surface-layer of the sediment was pneumatically removed following each sedimentation. The final lentil starch preparation, resulting from the previously described process, was dried in a laminar airflow tray-dryer/oven (APEX Construction LTD, Bromley, UK) and had a moisture and protein content of 11.30 ± 0.01% wt. and 0.85 ± 0.27% wt., respectively, determined by the gravimetric and Kjeldahl methods accordingly.

### 2.3. Film Preparation

The film preparation method consisted of casting the polymer solution in molds. Specifically, a 6.5% wt. starch suspension was prepared, to which STMP (at three different concentrations) and glycerol (30% wt. on dry starch weight) were added, and gelatinized at 80 °C for 30 min under continuous stirring. In the case of MMT addition, the solution was heated and stirred for 15 more minutes with an increase in the temperature by 10 °C. By the end of the essential heat treatment, a pH = 11 was achieved by adding a solution of 2 M NaOH to the mixture. Thereafter, 50 g of the mixture was transferred to a Teflon mold (18 cm × 11 cm × 1 cm (L × W × H)) and placed in a 50 °C oven for approximately 24 h. The composition of samples is provided in Table 1. In the case of MMT addition, a prior step was required, thus, the necessary quantity of the nanoclay was dissolved in distilled water and then submitted to stirring overnight. The next day, in order to be used as another ingredient of the film recipe, the MMT suspension was homogenized for 15 min with an ultrasonic processor (UP100H, Hielsher Ultrasonics GmbH, Germany). All films were conditioned for at least 7 days at room conditions (25 °C and 60% RH) prior to subjecting them to further characterization and analysis.

### 2.4. Thickness

Film thickness was measured using an electronic thickness gauge with an accuracy of 0.01 mm (MV40 86782, TESA, Renens, Switzerland). In order to have a complete picture of the thickness of each film, the measurements were taken at 9 random locations and mean values were calculated.

### 2.5. Moisture Content

Film moisture content was measured based on the method described by Galus et al. (2012) with some modifications [16]. Thus, water content was determined as the percentage of initial film weight loss after drying at 130 °C for 1 h.

### 2.6. Barrier Properties

Water vapor permeability rate (WVPR) was determined by a gravimetric method based on the method proposed by Mali et al. (2002) and the ASTM E96/E96M-05 desiccant method protocol (2005) with some modifications [17]. Each film sample was sealed over the circular opening of a permeation cell, which was stored at 35 °C in a desiccator. The relative humidity (RH) inside the desiccator and across the film was maintained at 75% with a saturated sodium chloride solution. To maintain 0% RH inside the permeation cell, silica gel was placed inside. Thus, water vapor transport was determined from the weight gain of the silica gel. After a 2 h equilibration, eight weight measurements took place at different time points during 48 h and changes in the cell weight were recorded to the nearest 0.001 g and plotted as a function of time. The slope of each line was calculated by linear regression (r^2^ > 0.99) and the water vapor transmission rate (WVTR) was calculated from the straight-line slope (g h^−1^), divided by the cell circular opening area (m^2^). Water vapor permeability rate (g Pa^−1^ h^−1^ m^−1^) was calculated as

(1) $$\mathrm{WVPR}=\frac{WVTR}{S(R_{1}-R_{2})}\;d$$

where S is the saturation vapor pressure of water (Pa) at the test temperature (35 °C), R_1_ the RH in the desiccator, R_2_ the RH in the permeation cell, and d the film thickness (m). Under these conditions, the driving force [S(R_1_ − R_2_)] was 3169.65 Pa.

### 2.7. Mechanical Properties

The mechanical properties of the films during tension were studied using a texture analyzer (TA.XΤ Plus, Stable Micro Systems, UK) based on the ASTM D882—10 standard test method for tensile properties of thin plastic sheeting (2012). These properties include elongation at break (%) and tensile strength (MPa). Each film was cut using a scalpel into rectangular strips 1.5 cm wide and 12 cm long, where 10 cm was the functional part, while 1 cm corresponded to each grip of the instrument. Pieces of rubber were placed between the edge of the film and the grip of the instrument to prevent unnecessary stress on the film. The texture analyzer head speed was set at 50 mm/min.

### 2.8. Color Properties and Opacity

Color properties were measured with a non-contact imaging spectrophotometer (MetaVue VS3200, Xrite, Grand Rapids, MI, USA). Additionally, the CIE lab values (L*, from black (0) to white (100); a*, from green (−128) to red (127); and b*, from blue (−128) to yellow (127)) were adopted to characterize the film color [18]. Film color images were reproduced by the IQC Color Software of the instrument based on the corresponding absorbance wavelength of each film. Film opacity index (cr) was measured with the same non-contact spectrophotometer that was also used for the determination of the color properties by measuring the over-light over dark (OLOD) feature on the IQC Color Software, which compares the absorbance of the standard black and white calibration plate in the absence and presence of the film.

### 2.9. Statistical Analysis

All statistical analyses were performed with Minitab 20 (Minitab Inc., State College, PA, USA). The homogeneity of variances and the normality of the residuals were tested. Once it was determined that the assumptions of the analysis of variance (ANOVA) were met for these data, the general linear model (GLM) procedure, with a fixed effect of treatment and a random effect of replication, was used for the statistical determination of all variables. Tukey’s and Fisher’s tests were used to determine the differences between the treatment means. Differences were considered statistically different at *p* < 0.05 and the results in each experiment were obtained by the mean values of two replicates from three independent experiments (total number of 6 analyzed samples).

## 3. Results and Discussion

### 3.1. Thickness

**Figure 1:**
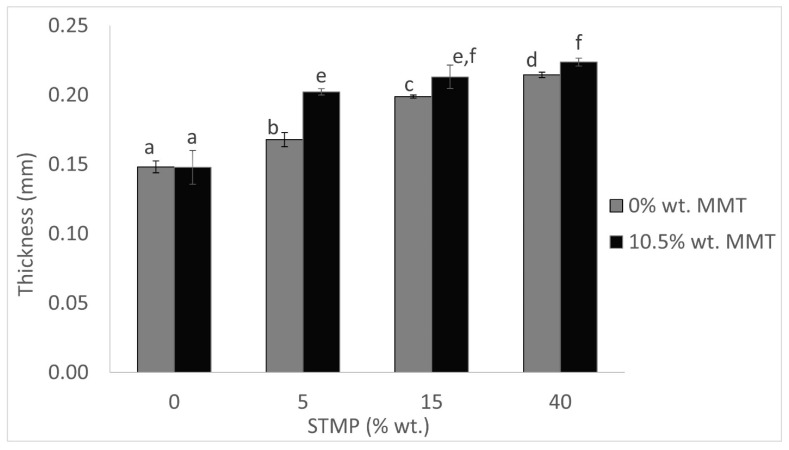
STMP crosslinking and MMT addition effect on film thickness. Data are presented as the average ± standard deviation. Groups that do not share a letter have a statistically significant mean difference (*p* < 0.05).

Film thickness is a substantial parameter for its barrier and mechanical properties. The film thickness ranged from approximately 0.15 mm to 0.22 mm. The thinnest films were non-crosslinked no matter if MMT was present (Sample E) or not (Sample A). The greater the concentration of the crosslinking agent was, the more the thickness was increased. The addition of STMP at concentrations 5, 15, and 40% wt. on dry starch increased the mass/area ratio, since the same containers for casting were used, and the increase in film thickness could be attributed to this fact. Adding MMT led to an extra increase in the thickness of the crosslinked films, as can be clearly seen in Figure 1. For instance, 5% wt. STMP increased the thickness from 0.15 mm (Sample A) to 0.17 mm (Sample B), while the sample with 5% wt. STMP and 10.5% wt. MMT (Sample F) reached a thickness of over 0.20 mm. A first explanation for the increased thickness observed after the addition of MMT is that the total amount of solids in the film was increased since MMT was added at a 10.5% wt. concentration based on dry starch weight. Another explanation could be that, due to the addition of MMT, less water is released and, because of surface dehydration, some of the water was entrapped within the polymer matrix and therefore resulted in thicker films [19]. Both STMP crosslinking and MMT addition contributed to the increase in the film thickness.

### 3.2. Moisture Content

**Figure 2:**
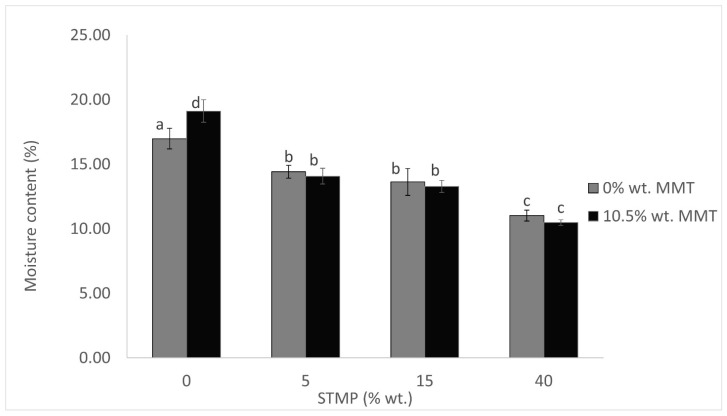
STMP crosslinking and MMT addition effect on film moisture content. Data are presented as the average ± standard deviation. Groups that do not share a letter have a statistically significant mean difference (*p* < 0.05).

Film moisture content determines the amount of water that was present in the film after its conditioning and ranged from 10.48–19.11%. According to Figure 2, the moisture content gradually decreased while increasing STMP concentration. The same observation was made by Sharma et al. (2020), who produced films with starch crosslinked with STMP, which suggests that the phosphorus group of STMP improved the chemical interactions in starch, and reduced the water that remained within the crosslinked films [5]. Nevertheless, in the present study, significant differences were not observed for small increases in STMP concentrations particularly from 5 to 15% wt. The moisture content was generally higher than that measured for films prepared from sorghum starch crosslinked with STMP and sodium tripolyphosphate (9.4%) [20], but lower than that measured for films prepared from faba bean starch crosslinked with STMP (19.16–24.08%) [5]. The addition of MMT did not seem to alter this property, even though Heydari et al. (2013) observed an increase in water content because of the addition of MMT [19]. Nevertheless, other studies show that the addition of MMT did not affect moisture content in accordance with our results [21]. The fact that the moisture content was not increased after MMT addition indicates that the aforementioned increase in film thickness could only be attributed to the increased total amount of solids in the film. In the case of film moisture content, it seems that the addition of MMT did not alter the effect of STMP crosslinking.

### 3.3. Barrier Properties

**Figure 3:**
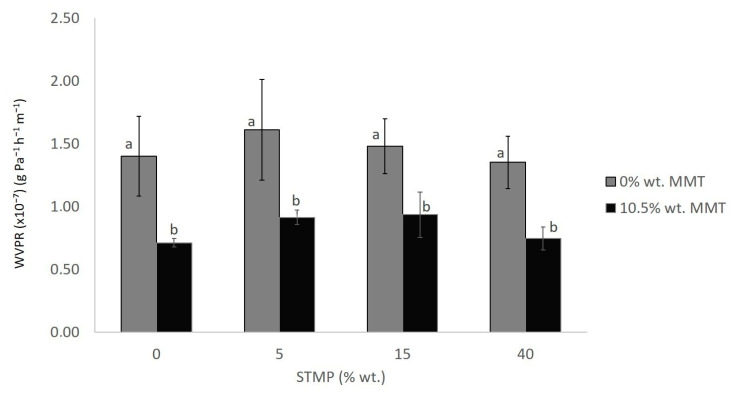
STMP crosslinking and MMT addition effect on film water vapor permeability rate. Data are presented as the average ± standard deviation. Groups that do not share a letter have a statistically significant mean difference (*p* < 0.05).

Water vapor permeability describes the ability of the film to prevent or facilitate moisture uptake. Usually, crosslinking agents improve the moisture barrier properties of films, since they react with the free hydroxyl groups present in starch and thus decrease their number, which makes the diffusion of water through the film more difficult [22]. However, STMP crosslinking did not have any effect on WVPR and the only observed statistical difference was due to the presence of MMT (Figure 3). The measured values are in good agreement with those of films prepared from rice starch crosslinked with STMP (0.92–2.60 g Pa^−1^ h^−1^ m^−1^) [23]. According to Vaezi et al. (2019), this trend in the decrease in the water vapor permeability is explained by the fact that the presence of montmorillonite particles forces the water vapor molecules to follow a longer and more tortuous route through the film matrix [4]. In the case of film barrier properties, it seems that MMT addition had a more prominent effect than STMP crosslinking.

### 3.4. Mechanical Properties

**Figure 4:**
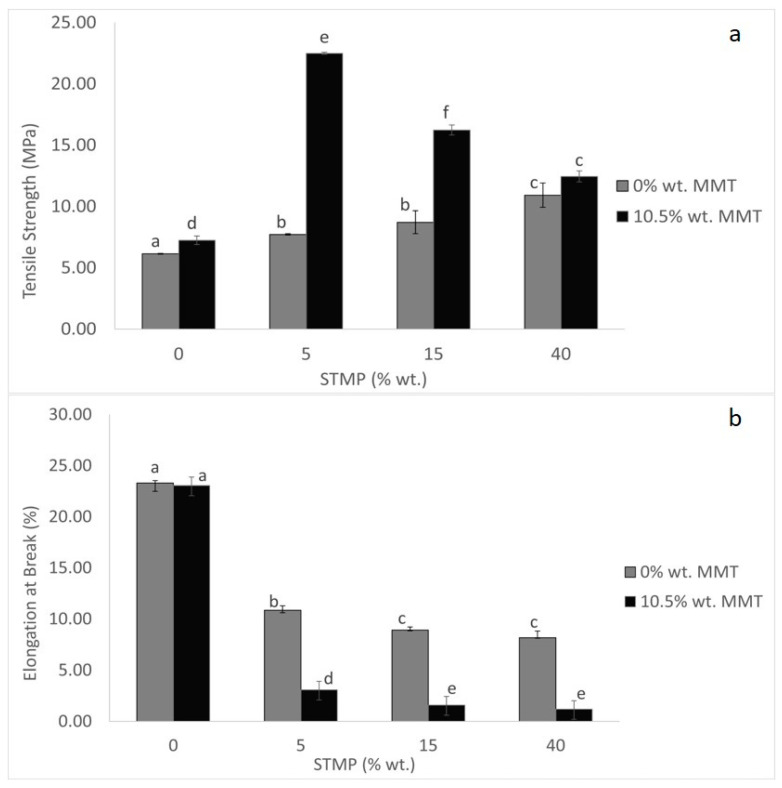
STMP crosslinking and MMT addition effect on film tensile strength (**a**) and elongation at break (**b**). Data are presented as the average ± standard deviation. Groups that do not share a letter have a statistically significant mean difference (*p* < 0.05).

The mechanical properties of starch-based films, tensile strength (TS), and elongation at break (EB), are the ones determining the ability of the packaging material to withstand external forces while maintaining its integrity. The TS of the film describes its ability to support maximum applied load without fracture. The non-crosslinked films without MMT (Sample A) showed the minimum TS, at 6.13 MPa, while the maximum TS was observed for the MMT-reinforced films with 5% wt. STMP (Sample F), at 22.49 MPa. Generally, in films where no MMT was added, the TS gradually increased as the percentage of STMP was increased (Figure 4a). This rise could be attributed to an increase in the crosslinking density of starch films that is caused by the reaction between the hydroxyl groups of starch and the crosslinking agent [24]. The observed values (6.13–10.92 MPa) were in good agreement with those observed for films prepared from faba bean starch crosslinked with STMP (8.25–14.28 MPa) [5] and rice starch crosslinked with STMP (~6.5–8.5 MPa) [23]. When MMT was added to the films, the TS values of 0, 5, and 15% wt. STMP concentrations were improved when compared with the corresponding ones where no MMT had been added. This effect could be attributed, according to Zoumaki et al. (2019), to the interactions between the plasticizer, the starch, and the montmorillonite during diffusion of glycerol molecules and starch chains inside the clay galleries [25]. However, regarding the effect of STMP crosslinking in the presence of MMT, something unexpected can be noticed: the TS peaked when the concentration of STMP was 5% wt. (Sample F), suggesting a synergistic effect, and then it dropped until no statistically significant difference could be observed between films crosslinked with 40% wt. no matter if MMT had been added, suggesting a negative interaction between STMP crosslinking and MMT addition. We consider that this could be attributed to steric hindrance effects caused by the presence of MMT, which hindered the formation of more crosslinks when excessive concentrations of STMP were added, due to the lack of available space in the matrix.

Elongation at break (EB) which indicates the flexibility of the film, as can be seen in Figure 4b, decreased as the amount of STMP increased. The same observation was noted by Detduangchan et al. (2014), which was attributed to the crosslinking reaction that limited chain movement while strengthening the starch chain [23]. Regarding findings from other researchers, measured values vary a lot ranging from 1.70% for films prepared from sorghum starch crosslinked with STMP and sodium tripoly-phosphate [20] to ~70% for films prepared from rice starch crosslinked with STMP [23]. Another point worth noticing is that the addition of the nanoclay led to a further decline of the elongation at break, since, according to Shayan et al. (2015), nanoclay on its own could cause a limited chain movement because of the interaction between hydroxyl groups in silicate layers with starch [26]. Eventually, the MMT-reinforced films were by far more brittle than the ones where no MMT had been added. Therefore, STMP crosslinking and MMT addition had a synergistic effect on the elongation at break. Both STMP crosslinking and MMT addition result in the formation of more junction zones than entanglements between the starch chains, making the film matrix more rigid and, consequently, increasing the tensile strength and decreasing the elongation at break.

### 3.5. Color Properties and Opacity

**Figure 5:**
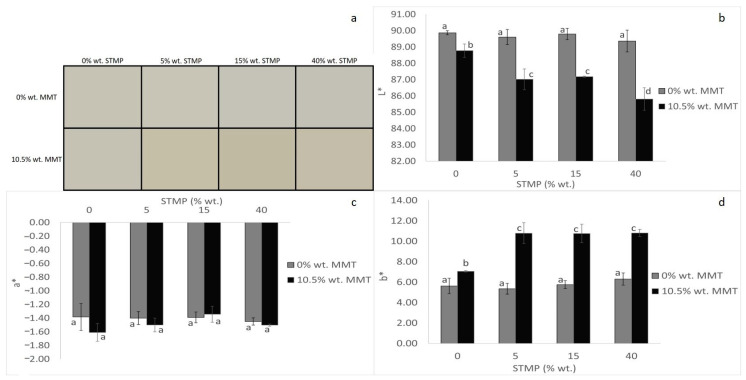
STMP crosslinking and MMT addition effect on film color (**a**), lightness (L*) (**b**), greenness (a*) (**c**), and yellowness (b*) (**d**). Data are presented as the average ± standard deviation. Groups that do not share a letter have a statistically significant mean difference (*p* < 0.05).

The color of the packaging is an important factor in terms of general appearance. Generally, crosslinkers change the color of starch films, and usually, the crosslinked films indicate decreased lightness (L*) and increased yellowness (b*) and greenness (a*) [27]. As can be seen in Figure 5, when no MMT had been added, STMP did not cause any significant alterations concerning the color of the films, despite the above-mentioned. In terms of other researchers’ findings, films prepared from corn starch crosslinked with STMP had an L* value of 91.82, a b* value of 11.64, and an a* value of −1.12 [28]. In the presence of MMT, however, increasing the STMP concentration caused a decrease in lightness (Figure 5b) and an increase in yellowness (Figure 5d), while greenness remained unaffected (Figure 5c). The addition of MMT resulted in a decrease in lightness (Figure 5b) and an increase in yellowness (Figure 5d), as was observed by Sothornvit et al. (2010) as well [29]. The greenness values did not differ statistically (Figure 5c), even when MMT was added, unlike what was stated by Sothornvit et al. (2010), who noted a linear drop with increasing clay content [29]. Therefore, STMP crosslinking and MMT addition had a synergistic effect only in the case of L* and b*.

**Figure 6:**
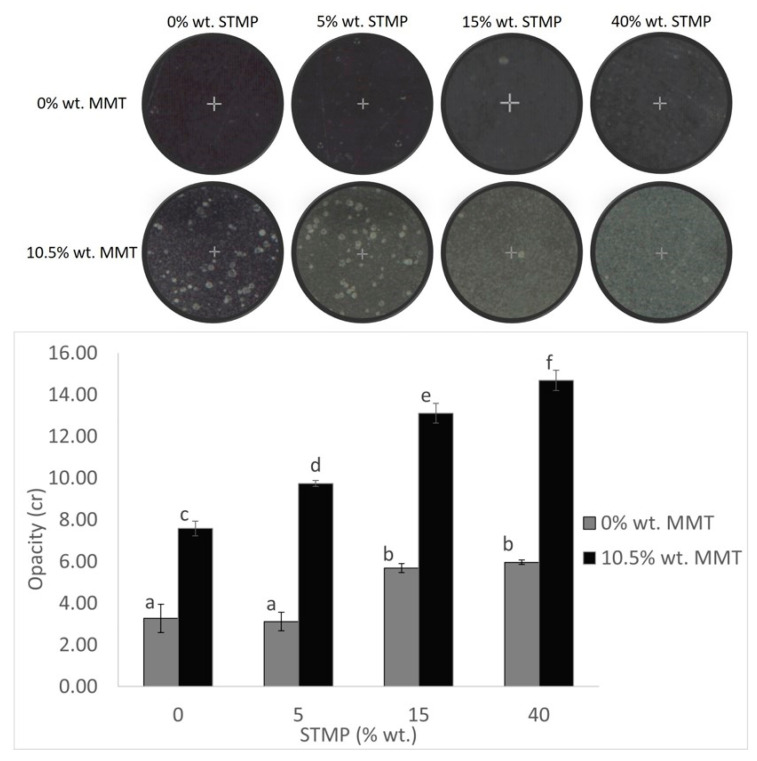
(**Up**) Photographs showing the STMP crosslinking and MMT addition effect on the film transparency. (**Down**) STMP crosslinking and MMT addition effect on the film opacity values. Data are presented as the average ± standard deviation. Groups that do not share a letter have a statistically significant mean difference (*p* < 0.05).

The transparency of the films is mostly important for consumer acceptance and it is commonly known that crosslinking affects this property. As shown in Figure 6, when no MMT had been added, the addition of 5% wt. STMP did not make the films any more opaque or transparent than the non-crosslinked ones. Similar opacity values (~2%) were measured for films prepared from faba bean starch crosslinked with STMP [5]. In contrast to the aforementioned films, the ones that were crosslinked with 15% and 40% wt. STMP presented higher opacity. Wu et al. (2019) also observed that the higher the crosslinking agent concentration was, the greater the opacity value and the less transparent the films were [30]. Li et al. (2019) ascribed these changes to the reaction between the crosslinker and the matrix, resulting in a more compact structure that hinders light transmission and therefore corresponds to a higher opacity [31]. An interesting fact is that the addition of MMT not only increased the opacity levels of the films in general but accentuated the effect of STMP crosslinking as well, making these films far blurrier than the films without MMT. As it has been argued by Sothornvit et al. (2010), the increase in the opacity when nanoclay was used in the film-forming blend, was mainly due to the aggregation of nano-particles which obstructs the transmission of light [29]. It is possible that crystallites were formed, whose size exceeded the wavelength of the incident light thus increasing the opaqueness of the film. This could explain why crosslinked films with MMT were the opaquest ones, since both MMT and STMP tend to increase the values of this property, showing once more a synergistic effect between them.

## 4. Conclusions

Experimental data revealed a synergistic effect between STMP crosslinking and MMT addition regarding most film properties. Therefore, the two methods of enhancing starch film properties (STMP crosslinking and MMT addition) can be successfully combined to produce films with better mechanical and barrier properties than when each method is applied on its own. The ability of the films to seal with starch glue renders them completely biodegradable and, in combination with the biocompatibility of all components, makes them an excellent choice for food packaging. The materials used to prepare the films are safe for human consumption; therefore, safety issues should not be a subject of major concern. These films are tested in our lab for storage of dry foods such as rice and legumes, while research is in progress for storing more perishable foods, such as minced meat in refrigerating and freezing conditions.

## Figures and Tables

**Table 1:** Description of the samples’ composition.

Code Name	STMP (% wt.) on Dry Starch Weight	MMT (% wt.) on Dry Starch Weight
A	0	0
B	5	0
C	15	0
D	40	0
E	0	10.5
F	5	10.5
G	15	10.5
H	40	10.5

## Data Availability

Data presented in this study are available on request from the corresponding author.
